# Dual Ectopic Thyroid: A Case Report with Review of Literature

**Published:** 2011-03-30

**Authors:** A. Sood, R. K. Seam, M. Gupta, D. Raj Sharma, P. Bhardwaj

**Affiliations:** 1 Assistant Professor, Nuclear Medicine Center, Department of Radiotherapy, Indira Gandhi Medical College, Shimla, India; 2 Professor, Department of Radiotherapy, Indira Gandhi Medical College, Shimla, India; 3 Assistant Professor, Department of Radiotherapy, Indira Gandhi Medical College, Shimla, India; 4 Professor, Department of ENT, Indira Gandhi Medical College, Shimla, India; 5 Assistant Professor, Department of Pediatrics, Indira Gandhi Medical College, Shimla, India

**Keywords:** Dual Ectopic Thyroid, Tc-99m Pertechnetate Scan, Subclinical Hypothyroidism

## Abstract

The ectopic thyroid gland is a rare entity which is mostly found along the line of descent of the thyroid gland. Most of the patients present with midline swelling and usually seek medical attention. Dual ectopic thyroid gland is even rarer. The clinical examination and different imaging modalities establish its diagnosis. Radionuclide studies are highly sensitive and specific in demonstrating the functional tissues in patients with ectopic thyroid, thereby guiding further management. The authors reported a case of ectopic thyroid gland in a girl with midline neck swelling initially, subsequently lost to follow-up. She again presented with enlarged swelling after a period of three years with dual ectopic thyroid in the neck region on thyroid scan. Thyroid scintigraphy demonstrated that progression in the size of ectopic glands was due to neglect in treatment.

## Introduction

The ectopic thyroid tissue (ETT) is a rare congenital anomaly defined as the thyroid tissue not located antero-laterally from the second to the fourth tracheal cartilage. An ectopic thyroid gland may occur anywhere along the path of initial descent of the thyroid with the majority of them in the midline neck region. The most commonly found ETT is at the base of the tongue, lingual thyroid accounting for 90% of reported cases.[1] Other rare sites for these tissues are from sublingual, higher cervical, intratracheal, mediastinal to below the diaphragm.[2] The exact cause of such locations is not known; however, it could be either because of development defects or aberrant migration of the gland. The ETT may be the only functioning thyroid tissue or may coexist as a separate structure with a normally located thyroid gland.[3] It is more unusual for two distinct foci of ETT to be present simultaneously. The web search of Pubmed in English literature revealed only 29 cases of dual ectopic thyroid so far, with the majority of them in the midline region of the anterior neck. We describe a case of a young patient of dual ectopic thyroid where the diagnosis and its subsequent management were established with the help of Tc-99m pertechnetate thyroid scan.

## Case Presentation

**Fig. 1:**
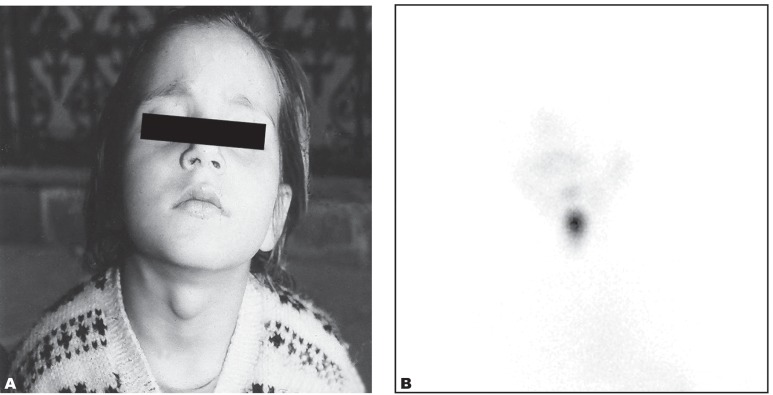
A 5-year-old girl with neck swelling.( A. Clinical photograph shows the midline neck swelling. B. Tc-99m pertechnetate thyroid scan shows a focus of abnormal radiotracer uptake in the subhyoid region and another very faint focal uptake in the sublingual region without any evidence of radiotracer uptake in the normal thyroid position in the anterior view.)

A five-year-old girl presented with asymptomatic midline swelling in the upper neck, gradually progressing in size (1.5×1 cm) with no feature of hypo- or hyperthyroidism in 2005. It was soft in consistency, non-translucent in nature and moved with deglutition on local examination (Fig. 1A). Ultrasonography of the neck revealed a well-defined hypoechoic mass of 1.6×1.0×2.0 cm size with flow in it on color Doppler in the subhyoid region and the absence of thyroid tissue in the normal position. A probable diagnosis of ectopic thyroid gland in the subhyoid region was made. The thyroid scan with 2mCi (74MBq) Tc-99m pertechnetate revealed a midline focus of tracer uptake in the subhyoid region suggestive of a subhyoid ectopic thyroid gland (Fig. 1B). The biochemical parameters were suggestive of subclinical hypothyroidism with T3-170 ng/dL (normal value: 60-185), T4-10.2 μg/dL (normal value: 4.8-12.0) and TSH-6.5 μIU/ml (normal value: 0.3-5.5). The patient was put on thyroxine, but she did not comply, being asymptomatic and was lost to follow up.

**Fig. 2:**
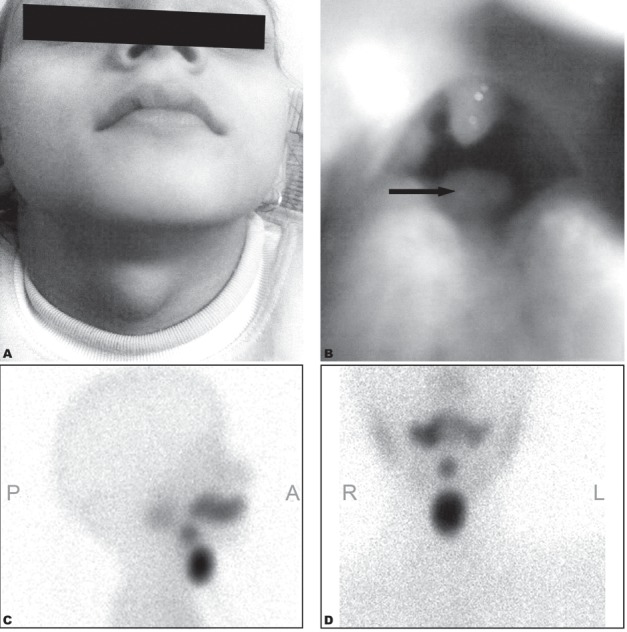
The same patient at the age of 8 years with neck swelling.( Clinical photographs show the midline neck swelling (A) and oral cavity swelling (arrow). (B) Tc-99m pertechnetate thyroid scan shows two foci of abnormal radiotracer uptake in the sublingual and subhyoid regions with no evidence of radiotracer uptake in the region of normally placed thyroid in lateral (C) and anterior views (D).)

She reported again after a period of 3 years with an increase in the size of the previous neck swelling, lethargy and constipation (Fig. 2A). On examination, the patient had another asymptomatic swelling located at the base of the tongue. This swelling was globular shaped, red in color and of 1×1 cm size (Fig. 2B). The patient was unaware of the oral swelling till her examination in the hospital. The ultrasound of the neck was suggestive of a similar diagnosis as given in 2005 (subhyoid ectopic thyroid gland), but with an increase in size to 2.2×1.4×2.4 cm. Repeated thyroid scan with 2 mCi of Tc-99m pertechnetate clearly revealed two distinct foci of tracer uptake in the midline neck in the sublingual and in the subhyoid area with a diagnosis of dual ectopic thyroid (Fig. 2 C and D). The ectopic thyroid in the subhyoid area was clearly enlarged in comparison to the previous one. The sublingual thyroid gland was not found in the initial scan carried out in 2005, probably it was small in size at that time and had very faint tracer uptake which was mistaken for tracer in the oral mucosa. However, it became prominent in the repeated scan performed after a period of 3 years. The present thyroid function tests revealed increased TSH values of 13.91, while T3 and T4 were within normal limits. The patient was started on thyroxine.

## Discussion

The ETT is seen at any age, but mostly noted at adolescence or after pregnancy due to the increased physiological demand of thyroid hormones.[1] Hormone production from ETT is usually insufficient, leading either to a subclinical or clinical hypothyroid state. It results into increased secretion of TSH from the pituitary gland in response to the hypothalmo-pituitary axis. Increased TSH level causes follicular cell hyperplasia of the ectopic thyroid tissue and visible swelling in front of the neck anywhere along the path of descent of thyroid primordium may be noted. The TSH stimulation may not have similar effect on different foci of ectopic thyroid tissue and they may have disproportionate growth.[4] Usually the patients complain of a palpable mass, growth retardation and lump sensation in the throat. Patients may have dyspnea, stridor or cough due to intratracheal thyroid gland. The incidence of clinical hypothyroidism with ETT varies from 24-60%. Adolescents and young adults may present with slow heart rate, chronic tiredness, cold intolerance, constipation, mental fatigue and difficulty in learning. Obesity, scholastic problems and delayed sexual maturation are other modes of presentation.[5] It is very rare for two distinct foci of ETT to be present simultaneously. Twenty-seven patients of dual ectopic thyroid have been reported in literature, reviewed in recently published articles by the author [6] and thereafter two more cases of dual ectopic thyroid were found on search in Pubmed.[7][8] The age range of these 30 patients (including the present case) was 4-71 years with a female to male ratio of 1.5:1. The patients were either asymptomatic or had an anterior neck swelling with or without altered thyroid dysfunction. Sixteen patients (53%) had swelling with a normal thyroid status, 11 (37%) had swelling as well as a hypothyroid status and two patients (7%) had swelling with hyperthyroid features, while the status of one patient (3%) was not available out of 30 patients. The majority of dual ectopies were shown to be in the neck and rarely away from the line of descent; the incidence at the lingual and sublingual sites was 21 and 8 out of 29 patients, respectively. The second ectopic thyroid tissue was seen at the subhyoid, suprahyoid, sublingual and porta hepatis region in 16, 9, 3 and 1 patients, respectively. The status of dual ectopic thyroid sites in one patient reported in the literature was not mentioned. Only two cases of dual ectopic thyroid tissue along with normally located thyroid gland have been reported in the literature.[9][10] In most of the cases, the diagnosis of dual ectopic thyroid was made on thyroid scan, though ultrasound and CT scan were additional imaging modalities in some of the cases.[6]

Lifelong thyroxine therapy is usually required according to individual thyroid status after establishing the diagnosis. It helps in achieving the euthyroid status as well as decreasing the size of ectopic thyroid swelling due to lowering of the elevated TSH level.[11] Surgery is usually not done, since the swelling may be the only functioning tissue in the body. It is only indicated if the patient is not relieved of pressure or obstructive symptoms, or there is suspicion of or proven malignancy in ETT.[12]

In our reported case, the patient did not have thyroxine which resulted into worsening of the thyroid function and increase in the size of ectopic glands in the subhyoid and probably in the sublingual region. This may be due to the increased physiological demand as the patient was entering the pubertal age group. This case highlights that the ectopic thyroid glands in patients even with subclinical hypothyroidism become enlarged due to neglect in treatment, as evident on clinical examination and scan findings. It also emphasizes the importance of replacement of thyroxine in such patients.
